# PPARS and Obesity

**DOI:** 10.1155/2007/78475

**Published:** 2007-06-07

**Authors:** Francine M. Gregoire, Sander Kersten, Wallace Harrington

**Affiliations:** ^1^Department of Biology, Metabolex, Inc., 3876 Bay Center Place, Hayward, CA 94545, USA; ^2^Division of Human Nutrition, Wageningen University, P.O. Box 8129, 6700 EV Wageningen, The Netherlands; ^3^Department of Metabolic Diseases, GlaxoSmithKline Research, 5 Moore Drive, Research Triangle Park, NC 27709, USA

Welcome to this special issue of PPAR Research dedicated to “PPARs and Obesity.” Obesity and the interrelated disorders of the metabolic syndrome are a global health epidemic. To address this major problem, it is essential to understand the mechanisms regulating energy metabolism, and it has been known for years that PPARs play an important role in many facets of energy homeostasis. This is a very active and exciting field of research that, without a doubt, justifies a special issue. The genetic, molecular, and physiological aspects of PPARs as well as the metabolic effects of recently developed PPAR and RXR agonists are among the topics discussed. The issue begins with a review of key observations made in human subjects harboring genetic variations in PPAR*γ* and a thorough overview of the metabolic effects of PPARs in genetically modified animal models. Over the last five years, the knowledge on PPAR delta biology has literally exploded and the potential therapeutic usefulness of this receptor in metabolic syndrome is now recognized. The interaction of PPARs with uncoupling proteins regulating energy expenditure is reviewed as are recent developments with RXR agonists. A closely related topic addresses the molecular and physiological functions of PPAR coactivators and corepressors in relationship to adipocyte energy metabolism. The selective PPAR modulator concept has attracted the attention of the field for over a decade; however the molecular bases underlying their differential mode of action have only begun to emerge recently. In addition to selective PPAR agonists, compounds that simultaneously activate two (dual agonists) or three (pan agonists) PPAR isoforms are in development. The potential advantages of these new combinations are discussed. The intriguing possibility that PPARs may mediate effects of caloric restriction on longevity is also considered. Finally, a growing body of evidence indicates that inflammation is a key feature of the obese state and that PPARs display strong anti-inflammatory properties. The evidence that PPARs may be interesting therapeutic targets to modulate obesity-induced inflammation is also reviewed. While these reviews just scratch the surface of PPAR/RXR interactions and regulation of energy balance, this special issue is packed with exciting, high quality reports from recognized experts in the field. We hope that the ideas presented here will generate further interest from the scientific community in this rapidly expanding area of research. 

*Francine M. Gregoire* *Francine M. Gregoire*

*Sander Kersten* *Sander Kersten*

*Wallace Harrington* *Wallace Harrington*

